# Roles and new Insights of Macrophages in the Tumor Microenvironment of Thyroid Cancer

**DOI:** 10.3389/fphar.2022.875384

**Published:** 2022-04-11

**Authors:** Qi Liu, Wei Sun, Hao Zhang

**Affiliations:** Department of Thyroid Surgery, The First Hospital of China Medical University, Shenyang, China

**Keywords:** thyroid cancer, cancer stem cell, tumor microenvironment, tumor associated macrophages, cytokines

## Abstract

Although most thyroid cancers have a good and predictable prognosis, the anaplastic, medullary, and refractory thyroid cancers still prone to recurrence and metastasis, resulting in poor prognosis. Although a number of newly developed targeted therapies have begun to be indicated for the above types of thyroid cancer in recent years, their ability to improve overall survival remain hindered by low efficacy. As the largest component of immune cells in tumor microenvironment, tumor-associated macrophages play a key role in the invasion and metastasis of thyroid cancer. There is much evidence that the immune system, tumor microenvironment and cancer stem cell interactions may revolutionize traditional therapeutic directions. Tumor-associated macrophages have been extensively studied in a variety of tumors, however, research on the relationship between thyroid cancer and macrophages is still insufficient. In this review, we summarize the functions of tumor-associated macrophages in different types of thyroid cancer, their cytokines or chemokines effect on thyroid cancer and the mechanisms that promote tumor proliferation and migration. In addition, we discuss the mechanisms by which tumor-associated macrophages maintain the stemness of thyroid cancer and potential strategies for targeting tumor-associated macrophages to treat thyroid cancer.

## Introduction

In recent years, thyroid cancer (TC) has attracted more and more attention due to its rapid increase in incidence. TC ranks the fifth in the incidence of all malignant tumors and has become the most common endocrine tumor (Cabanillas et al., 2016). It is important to note that the incidence of TC is also increased in children and adolescents (Person et al., 2021). The incidence of TC continues to increase not only because of environmental pollution, radiation exposure and other external factors, but also because of the improvement of imaging or ultrasound diagnostic technology and biopsy diagnosis (La Vecchia et al., 2015). Papillary thyroid carcinoma (PTC) and follicular thyroid carcinoma (FTC) account for 80%–85% and 10%–15% of TCs respectively (LiVolsi, 2011; Sowder et al., 2018). Patients with these differentiated TCs can benefit from surgery, radioiodine therapy, and thyroid stimulating hormone (TSH) inhibition therapy, and thus have a better prognosis. In contrast, medullary thyroid carcinoma (MTC) is one of the relatively rare thyroid malignant tumors, accounting for 1%–2% of all TCs, but it has a worse prognosis with a 10-year overall survival (OS) of only 81.2% (Cabanillas et al., 2016; Kotwal et al., 2021). Notably, there are approximately 2% of anaplastic thyroid cancer (ATC) (Smallridge et al., 2012), though rare, is often associated with resistance to radioiodine treatment and is characterized by an aggressive phenotype and poor prognosis, with an average survival time of few months (Cabanillas et al., 2016; Veschi et al., 2020). Currently, the clinical treatment for ATC is a great challenge.

Macrophages are highly plastic cells with multiple functions and these cells originate in the bone marrow from myeloid derived progenitor cells (Varol et al., 2015). Tumor-associated macrophages (TAMs) are derived from tumor-infiltrating monocytes in peripheral blood. Current studies indicate that TAM population is in a constant transition between M1 and M2 type (Pan et al., 2020). M1-macrophages have antitumor effects, through the identification of tumor cells and finally kill tumor cells, the study revealed M1-macrophages by two different mechanism of killing tumor cells, one is mediated cytotoxicity to kill tumor cells directly, and the other is antibody dependent cell-mediated cytotoxicity to kill tumor cells. M2-macrophages are divided into multiple subtypes, M2a is involved in tissue fiber formation, M2b contributes to tumor progression, M2c is responsible for tissue remodeling, and M2d promotes angiogenesis (Wang et al., 2019a). TAMs can promote tumor progression by secreting cytokines, such as vascular endothelial growth factor (VEGF) or fibroblast growth factor (FGF) (Mantovani et al., 2002). Therefore TAMs promote angiogenesis and are often associated with high density of blood vessels (Coffelt et al., 2010). In TCs, TAMs mainly exhibit the M2 phenotype (Solinas et al., 2010). Therefore, how to control the phenotypic transformation of TAMs may be the focus of future research. These cytokines or chemokines originate from cancer cells and macrophages, but the mechanisms by which these cytokines or chemokines act on tumors have not been fully elucidated. TAMs maintains the survival, proliferation and invasion of cancer cells and may be a potential therapeutic target (Bolli et al., 2017; Mantovani et al., 2017; Cassetta and Pollard, 2018).

Tumor microenvironment (TME) is mainly composed of tumor cells, innate and adaptive immune cells, fibroblasts, vascular, cytokines and chemokines, et al. TME can not only provide a suitable environment for tumor to promote tumor growth and metastasis, but also inhibit the occurrence and development of tumor through changes in metabolism, secretion, immunity and other conditions (Ben-Baruch, 2006; de Visser et al., 2006). TME is a complex and interactive network regulated by immune cells, cytokines and other elements. Tumor cells are regulated by their own genes and external factors, which jointly promote tumor development and phenotypic changes, such as EMT of tumor cells (Pradella et al., 2017; Weng et al., 2019). TC cells can maintain growth by secreting a variety of cytokines and also by secreting chemokines to recruit different immune cells (Lumachi et al., 2010; Fozzatti and Cheng, 2020). These cytokines or chemokines affect the progression of TC and changes in the immune microenvironment (Menicali et al., 2020). Innate immune cells, especially natural-killer cells (NK cells) and macrophages play a key role in the regulation of tumor progression and inhibition (Goldberg and Sondel, 2015). Similarly, these immune cells can also secrete some cytokines and angiogenic factors to remodeling the TME of TC (Poschke et al., 2011). In addition, tumor progression can also destroy the balance of the original microenvironment, making it conducive to tumor proliferation and migration. Immune surveillance is the function of the immune system to identify, kill and remove mutated cells from the body to prevent tumor development. Tumor immune escape is a phenomenon in which tumor cells evade recognition and attack by the immune system through various mechanisms, thus allowing them to survive and proliferate *in vivo* (Prendergast et al., 2010). Although immune cells tend to kill tumor cells in the early stage of tumor genesis, they seem to evade immune surveillance eventually, and even inhibit the cytotoxic effect of immune cells on tumor through various mechanisms (Lei et al., 2020).

At present, it is believed that TC is closely associated with the occurrence and development of inflammation, and about 20% of PTC is caused by chronic thyroiditis (Fugazzola et al., 2011). Chronic inflammatory is an important factor mediating tumorigenesis (Noy and Pollard, 2014). The original concept of “inflammation causes tumors” is gradually changed to “inflammation causes tumors, and tumors cause inflammation (Allavena et al., 2011).” In this review, we discuss the types of TAMs and their effects on TCs, the interaction between cytokines and chemokines in TME, cancer stem cell (CSC), and new strategies for immunotherapy of TCs.

## Tumor-Associated Macrophages in the Thyroid Tumor Microenvironment

**FIGURE 1:**
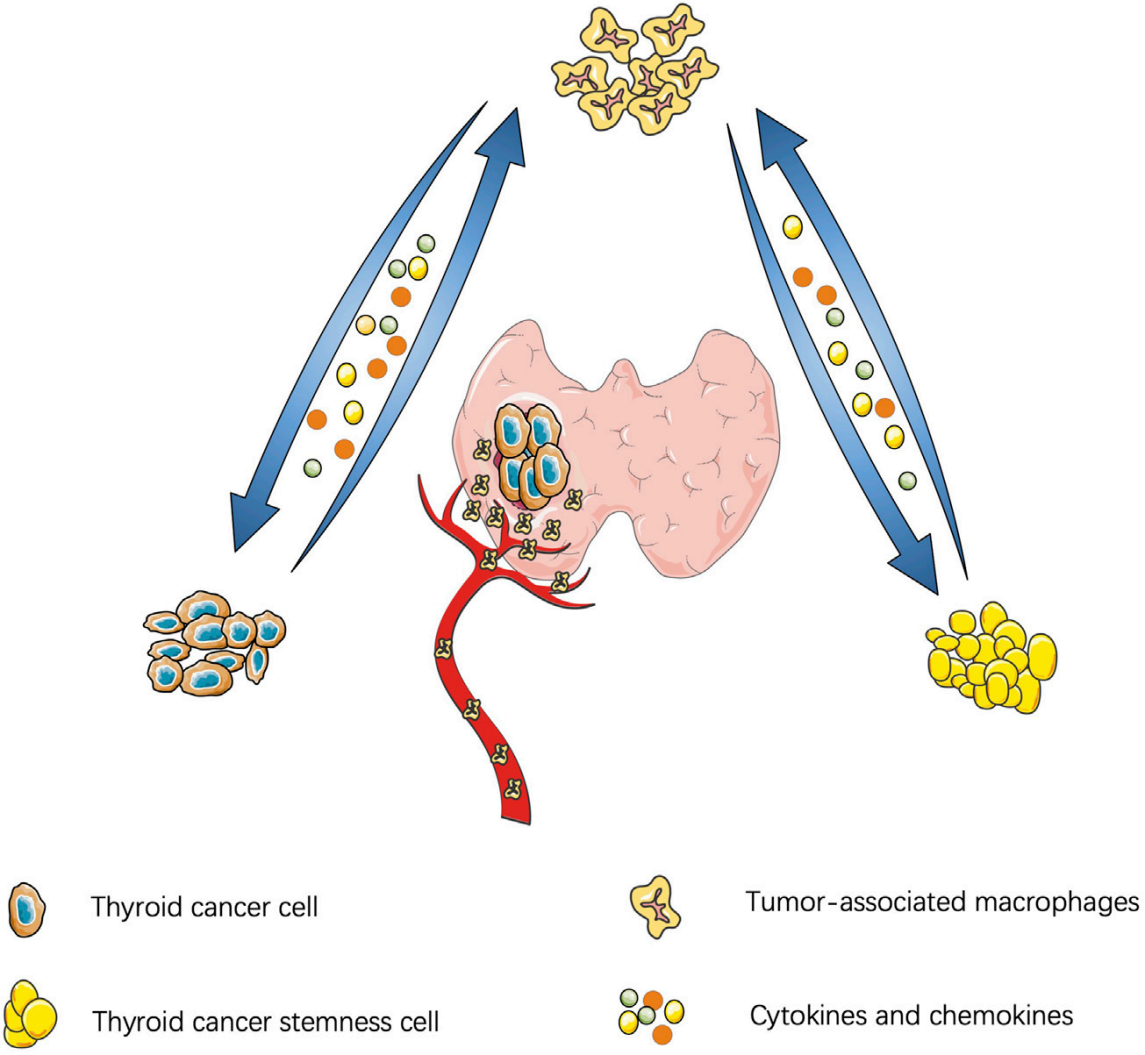
Interaction between thyroid cancer and tumor-associatated macrophages: Thyroid cancer cells secrete cytokines to manitain the stemness of cancer cells and cancer stem cells feedback to promote tumor proliferation and migration. Thyroid cancer can also secrete chemokines to recruit macrophages and polarize into M2 subtype. Similarly, cytokines derived from tumor-association macrophages promote tumor migration.

Similar to other malignant tumors, the thyroid-TME is composed of immune cells (macrophages, mast cells, neutrophils, and lymphocytes) and soluble mediators (chemokines, cytokines, and growth factors) that are active in and around cancer cells (Yapa et al., 2017). Currently, TAMs infiltration in TME is believed to be through recruitment of circulating monocytes. Cytokines and chemokines in TME promote polarization of macrophages and affect the functions of TAMs, such as promoting tumor proliferation, stemness, gene instability, blood vessel and lymphatic proliferation, immunosuppression, et al. (Mantovani and Allavena, 2015). A schematic diagram of the interaction between TC and TAM is shown in Figure 1.

TC is commonly associated with a complex genetic background and gene mutations, that could be involved in mutations in BRAF, RAS and PI3K signaling pathways. Losing or inhibiting the functions of PTEN, p53 and B-catenin is commonly seen in poorly differentiated TCs (Hou et al., 2007). BRAF^V600E^ mutation is most frequent in PTCs or ATCs (Cancer Genome Atlas Research, 2014). While RAS mutation mostly exist in FTCs and other variant of PTCs (Cabanillas et al., 2016). RET proto-oncogene mutation is thought to be the cause of most MTC, while a low proportion of TCs is caused by sporadic RAS mutation (Ciampi et al., 2013; Wells et al., 2015). In an *in vivo* study of nude mice, high CXCL16 expression is associated with M2-macrophage infiltration in BRAF^V600E^ mutated PTC, promoting tumor angiogenesis and resulting in poor prognosis (Kim et al., 2019). In another study, In BRAF^V600E^ and BRAF^WT^, the average ratio of immune cell populations CD68 +/CD163 + cells tends to decrease, resulting in extensive immunosuppression of TC in BRAF^V600E^ (Angell et al., 2014).

### Polarization of Tumor-Associated Macrophages in Tumor Microenvironment

Macrophages recruited from circulating monocytes into tumor tissues and endowed with tumor-promoting or suppressive effects are called TAMs (Pathria et al., 2019; Cassetta and Pollard, 2020). Once TAMs from peripheral blood monocytes are recruited to TME by tumor secreted chemokines and polarized into M1/M2 macrophages under various stimuli. TAMs can also show significant plasticity within the TME, transforming from one phenotype to another in response to certain stimuli (Sica and Mantovani, 2012).

M1-TAMs are involved in the activation of Th1 type immune response and they have a high capacity of antigen presentation, secreting some cytokines and chemokines such as IL-1β, IL-6, IL-12, IL-23, CXCL9, CXCL10, TNF-α, etc., (Porta et al., 2015; Wu et al., 2020). In addition, Some surface proteins of M1-TAMs, CD68, CD80 and CD86, are also upregulated (de Sousa et al., 2016). In contrast, M2-TAMs primarily upregulated the expression of anti-inflammatory cytokines and chemokines, including IL-10, TGF-β, CCL17, CCL18, CCL22, and CCL24. Similarly, some surface proteins of M2-TAMs are upregulated including CD163, CD204 and CD206 (Biswas et al., 2013; Jayasingam et al., 2019).

**FIGURE 2:**
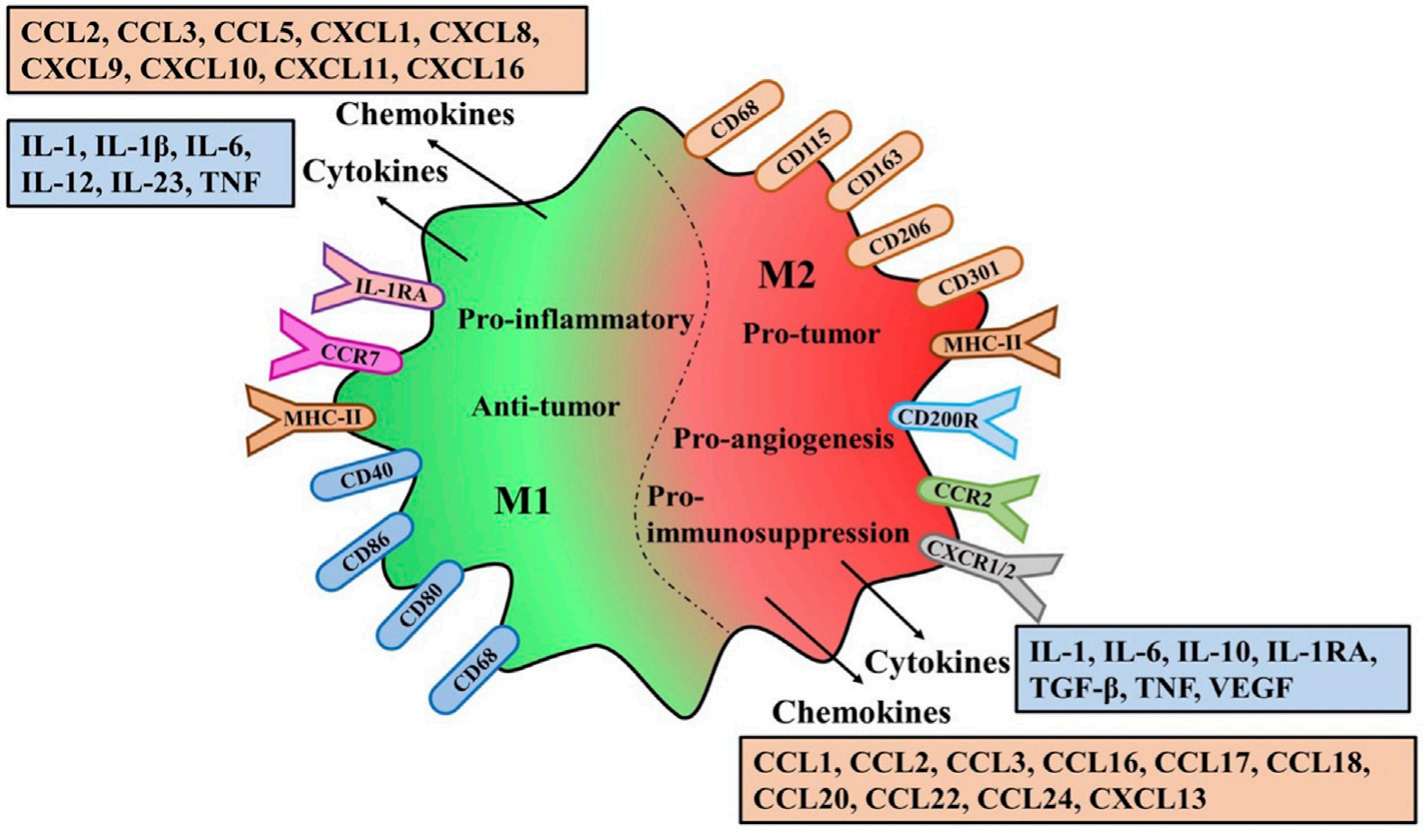
Polarization characteristics surface markers receptors and secretions of TAMs.The variation of M1/M2-TAMS in the Tumor microenviroment the same or different markers and receptors on the membrane surface are explained. Different types of Polarization secreted different cytokines and chemokines and different effects on tumors

Although many markers related to TAMs polarization have been discovered, some markers of M1 or M2 phenotype can be co-expressed on an individual cell (Chong et al., 2015). Therefore, the anti-tumor or pro-tumor effects of M1 or M2 macrophages are still controversial. Their effects can be determined not only by the high and low expression of markers, but also by the complex activation mechanism of the mutual transformation between the two phenotypes (Figure 2).

### Roles of Tumor Associated Macrophages in Tumor Microenvironment

Hypoxia is an effective means to induce TME to recruit TAMs, and imbalance of TME oxygen supply may inhibit TAMs migration. Hypoxia inducible factor-1α (HIF-1α) is a key transcription factor regulating hypoxia-induced gene expression, and its high expression can induce TAMs to enter hypoxia region continuously. HIF-1α upregulates CXCR4 and its ligand CXCL12 in monocytes/macrophages and induces these chemotactic responses (Kumar and Gabrilovich, 2014).

After recruitment and polarization, M2-TAMs can secrete a series of cytokines to stimulate tumor cell proliferation and survival including epithelial growth factor (EGF), epithelial growth ligands of the factor receptor (EGFR) and basic fibroblast growth factor (bFGF) (Yin et al., 2016). Polarization of TAMs has been confirmed to be associated with proliferation and metastasis of various malignant tumors. In ovarian cancer, TAMs can promote tumor proliferation and migration by upregulating insulin like growth factor (IGF1) signaling pathway (Liu et al., 2018a). Gastric cancer derived mesenchymal cells secrete IL-6 and IL-8 (CXCL8) through JAK2/STAT3 signaling pathway and induce polarization of gastric cancer M2-macrophages. M2-macrophages significantly promote gastric cancer metastasis through promoting epithelial-mesenchymal transition (EMT) mechanism (Li et al., 2019). In a study on exosomes and TME, Mir-183-5p level of M2-TAMs exosomes was significantly increased, which targeted THEM4-mediated Akt/NF-κB and inhibited the mRNA and protein expression, promoted the proliferation and invasion of colon cancer cells, and inhibited apoptosis. Downregulation of Mir-183-5p reversed the M2-TAMs mediated tumor promotion (Zhang et al., 2021a). In some clinical studies of non-small cell lung cancer, M2-TAMs infiltrate induce tumor cell invasion and progression, resulting in poor prognosis (Sumitomo et al., 2019; Sedighzadeh et al., 2021). Pulmonary macrophage accumulation is one of the important factors leading to lung metastasis of ATC (Li et al., 2016). Recent studies have found that some gene mutations can cause imbalance of M1/M2-TAMs. For example, a recent animal study revealed that Mettl3-deficient mice showed increased infiltration of M1/M2-TAMs. Deletion of METTL3 disrupts YTHDF1-mediated translation of SPRED2, thereby enhancing NF-kB and STAT3 activation through the ERK pathway, leading to increased tumor growth and metastasis (Yin et al., 2021). When the mTOR pathway is activated, the monocyte macrophage phenotype is converted to M2-TAMs, enhancing pro-angiogenic capacity. On the contrary, when the mTOR pathway is inhibited, the cell phenotype is transformed into M1-TAMs, and its decreased angiogenesis ability can inhibit tumor growth (Li et al., 2020). Therefore, under the structure of TME, M2-TAMs can not only secrete cytokines or chemokines to induce tumor development, but also promote tumor proliferation and migration through cross talk with tumor cells.

There is substantial evidence that inflammatory responses at tumor sites can promote tumor growth and progression. Inflammation and immune evasion are considered to be hallmarks of cancer.

### Interaction of Tumor-Associated Macrophages With Thyroid Cancer Stem Cells

CSC is a subpopulation of cancer cells with the ability to self-renew and proliferate heterogeneous cancer cells. CSCs have an important role in tumor survival, proliferation, metastasis, drug resistance and recurrence (Ayob and Ramasamy, 2018). CSCs are primarily found inside tumors, named stem cell niches, under specific microenvironmental conditions, that consist of multiple types of stromal cells, including the vascular system, mesenchymal cells, immune cells, extracellular matrix, and cytokines (Borovski et al., 2011). In 2005, Takano and his colleagues proposed that TC cells were derived from stem cells or precursor cells of fetal origin in normal thyroid gland rather than from differentiated thyroid follicular cells.

Based on this hypothesis, many scholars believe that TC is a CSC-driven disease that may originate from the transformation of stem cells or from the dedifferentiation process of cancer cells (Zhang et al., 2006; Zito et al., 2008). CSCs are predominantly found in different specific TME, where the dynamic balance between intracellular and extracellular cytokines produced by the TME allows the maintenance of a stem cell phenotype characterized by a lack of tissue-specific differentiation, slow cell cycle rate, quiescence and a theoretically unlimited capacity for self-renewal (Pietras et al., 2014; Chan et al., 2019; Grassi et al., 2021). A growing number of studies have shown that CSCs are closely associated with radiotherapy tolerance, tumor metastasis and recurrence (Magee et al., 2012; Mertins, 2014). The transition of stem cells to differentiated cancer cells is stimulated by growth factors and cytokines that are present in the TME in which the stem cells are located (Gianì et al., 2020). TAMs secretes multiple cytokines or chemokines to support self-renewal and maintenance of stemness. Similarly, CSCs secrete pro-tumor signals to activate TAMs, which further promotes tumor development (Sainz et al., 2016). The expression of EMT biomarkers was strongly correlated with the presence of CSCs in TC and the role of TAMs (Lan et al., 2013; Hardin et al., 2018). CSCs are currently thought to drive TC heterogeneity, contributing to their metastatic potential and therapeutic resistance (Grassi et al., 2021). CSCs can also secrete exosomes to regulate the polarization of TAMs toward the M2 phenotype, inhibit NK-cell activity, and promote suppression of the immune microenvironment in CSC niches (Caruso Bavisotto et al., 2019; Baig et al., 2020). The current view is that eradication of CSCs may inhibit tumor recurrence, while failure to completely remove CSCs eventually leads to tumor recurrence (Nguyen et al., 2012). Although current research is limited, there is evidence that TAMs can enhance the stemness of CSC (Chanmee et al., 2014). In a study of head and neck cancer, TAMs can affect CD44 signal through PI3K-4EBP1-SOX2 pathway to mediate stemness enhancement, and *In vivo* experiments, Targeting CD44 reduced PI3K-4EBP1-SOX2 signal, inhibited tumor growth, and attenuated stemness (Gomez et al., 2020). Periostin secreted by glioma stem cells recruited peripheral blood mononuclear cells into glioblastoma tissue. Moreover, TAMs and glioma stem cells co-distribute in perivascular niches to maintain the characteristics of M2 macrophages and secrete tumour-supportive factors to promote glioblastoma growth and progression (Zhou et al., 2015).

Although the current research results on thyroid CSCs and TAMs are still insufficient. However, existing studies can show that there is a mutual promotion between thyroid CSCs, TAMs and TME, that is, TAMs can maintain the stemness of thyroid CSCs, and CSCs can also promote polarization of TAMs to M2 macrophages. Finally, CSCs and TAMSs establish a positive loop by a cross talk mechanism.

## Functions and Mechanisms of Macrophages in Thyroid Cancer

TAMs accounts for a large proportion of tumor-infiltrating immune cells in TME and are highly plastic (Chen et al., 2019). TAMs are infiltrating immune cells that can occupy up to 50% of the total volume of thyroid tumors (Ryder et al., 2008). TAMs respond to cytokines or chemokines secreted by tumor cells and polarize into M1 or M2-TAMs. but in TME, M2-TAMs are usually predominant. M1-TAMs play a key role in killing tumor cells by producing reactive oxygen/nitrogen species (ROS/RNS) and pro-inflammatory cytokines such as IL-1β, IL-6, and TNF-α, and thus M1-TAMs are macrophages with anti-tumor effects (Jeannin et al., 2018; Chen et al., 2019). Typically, most TAMs are M2-TAMs with a Th2 immune response, which can enhance tumor cell immune escape (Galdiero et al., 2013; Ferrari et al., 2019).

TC cells and M2-macrophages can promote each other. M2-TAMs promotes TC dedifferentiation, proliferation and metastasis by secreting Wnt1 and Wnt3 ligands, that activate the Wnt signaling pathway and promote β-catenin activation (Lv et al., 2021a). Inhibition of the Akt-mTOR signaling pathway inhibits TC-induced cytokines production by macrophages (Sloot et al., 2019a). Mazzoni et al. found that TC cells activate PGE2 secretion to promote M2 polarization, enhancing tumor invasiveness (Mazzoni et al., 2019). On the contrary, TCs can induce macrophages polarization, which in turn acts on tumor cells to lead to tumor progression and metastasis. For example, senescent thyroid tumor cells can induce M2-macrophages polarization with increased expression of CCL17,CCL18,IL-18,TGFβ1, that ultimately promotes tumor migration by activating NF-κB signaling pathway (Zhang et al., 2021b). In addition, gene mutations also have an impact on TAM polarization, such as BRAF^V600E^ mutation, which can lead to an increase in the proportion of M2-TAMs and promote tumor growth (Ryder et al., 2013). Some drugs or compounds can alter polarization of M2. For example, a recent study by Juan LV et al. found that Zoledronic acid inhibits M2-induced TC proliferation, stemness and metastasis by inhibiting M2 polarization and suppressing the Wnt/β-catenin signaling pathway (Lv et al., 2020). TNF-α derived from TAMs can induce IL-32β expression in TC cells. Although IL-32β does not directly affect TC cells migration, alternative splicing of IL-32 towards the IL-32β isoform may be beneficial for TC cell survival through induction of the pro-survival chemokine IL-8 (CXCL8) (Sloot et al., 2019b).

### M1-Macrophage Transformation in Thyroid Cancer

M1-macrophages can inhibit tumor proliferation and migration in a variety of malignant tumors. However, due to the limitations of various factors and conditions, studies on TC are still insufficient. Reversing the polarization of M2-macrophages has also emerged as one of the potential new strategies for TC treatment. Metabolic reprogramming of M1-macrophages has been found in autoimmune diseases to enhance glycolysis and inhibit oxidative phosphorylation, which may be one of the clues of autoimmune thyroiditis (Murray et al., 2014).

In PTC, the normal thyroid tissue had higher density of M1-macrophages than the cancer tissue. And the proportion of M1-macrophages in stage I and II is higher than that in other stages (Xie et al., 2020). More importantly, CIBERSORT results showed that some anti-tumor immune cells such as M1-macrophages, plasma cells and CD8^+^ T cells were positively correlated. Similarly, A the cancer genome atlas (TCGA) prognostic model of TC revealed that a higher proportion of M1-macrophages and dendritic resting cells in the low-risk group (Zhang et al., 2021c). In addition, the endocrine system is also involved in regulating the polarization of macrophages. *In vitro* results showed that triiodothyronine (T3) had a negative effect on triggering the differentiation of bone marrow derived monocytes into nonpolarized macrophages. M1-macrophages polarization of macrophages induced by T3 (Perrotta et al., 2014). *In vitro* experiments, the co-culture system of TPC-1 cells and bleomycin revealed that low-dose bleomycin could transform M2-TAMs into M1-TAMs, and the proliferation, migration and invasion abilities of TPC-1 cells were significantly reduced (Liu et al., 2018b). Another *in vitro* study found that in Graves disease (GD), activated NK-cells drive macrophages to differentiate to M1 phenotype, which in turn is cytotoxic to cancer cells and downregulates the M2 phenotype (Imam et al., 2019). This will open up new prospects for immunotherapy of TC. Macrophage/innate immunity can modulate from M2 phenotype to M1 phenotype to help treat TC as naturally done by GD.

### Functions of M2-Macrophages in Papillary Thyroid Cancer

The most common route of metastasis for PTC is lymphatic metastasis. Current studies have found that PTC can achieve the purpose of macrophage recruitment in TME by upregulation of some chemokine transcription programs (Guarino et al., 2010; Muzza et al., 2010). Previous studies have demonstrated that high density of M2-TAMs leads to lymph node metastasis in PTC (Gulubova and Ivanova, 2019). It was reported that M2 macrophages accumulated around the lymphatic vessels at the PTC tumor margins implicated lymphatic invasion of cancer cells (Kabasawa et al., 2021). M2-TAMs around lymphatic vessels enhance lymphatic invasion by upregulating MMP2. Yang et al. found that Mir-324-5p/PTPRD/CEBPD axis induces Human Umbilical Vein Endothelial Cells (HUVEC) invasion/migration and M2- macrophages polarization through VEGF and IL4/IL13, respectively, and participates in the progression of PTC (Yang et al., 2020a). TAMs conditioned medium co-cultured with PTC cell enhanced the invasion of PTC. More importantly, this study found that CXCL8 promotes PTC metastasis *in vivo*, confirming that TAMs may promote PTC metastasis through the interaction between CXCL8 and its receptor, CXCr1/2 (Fang et al., 2014). In addition, other cytokines secreted by TAMs also promote the invasion and metastasis of PTC. For example, The combination of CXCL16 derived from M2-TAMs with angiogenic genes leads to high aggressiveness of PCT, and knockout of endogenous CXCL16 delayed tumor growth in athymic mice (Cho et al., 2016). M2-macrophages also have immunosuppressive function, which can mediate immune escape and promote the proliferation and metastasis of TC. M2-TAMs promote the proliferation and metastasis of TC through a variety of paracrine methods. In addition, the upregulated level of VEGF and EGF secreted by M2-macrophages can promote tumor microangiogenesis by recruiting endothelial cells (Chandler et al., 2019). Hypoxia is also a major driver of tumor angiogenesis, and a large number of TAMs have been found in hypoxia areas of tumors, especially necrotic tissues (Yang et al., 2020b).

### Functions of M2-Macrophages in Anaplastic Thyroid Cancer

The density of M2-macrophages varied among thyroid tumors, with higher density in PTC and highest in ATC. The density of M2-macrophages is closely associated with tumor prognosis (Jung et al., 2015; Ferrari et al., 2019). The presence of a high density of macrophages in ATC, which are dominated by polarized M2-TAMs, is associated with tumor aggressiveness, and therefore M2-TAMs can be used as a biomarker to predict prognosis (Sica et al., 2006; Caillou et al., 2011). However, the high density of TAMs aggregation is not sufficient to evaluate the prognosis. It is critical to evaluate their function and signaling pathways (MacDonald et al., 2020). The infiltrating cells of ATCs include lymphocyte infiltrate (mainly CD8^+^ cytotoxic T cells) and TAMs (Cameselle-García et al., 2021; Prete et al., 2021). M2-TAMs can secrete insulin-like growth factors (IGF) to activate IR-A/IGF1R and mediate PI3K/AKT/mTOR signaling pathway to accelerate ATC cells metastasis and enhance stem cell viability and stemness (Lv et al., 2021b). A recent study revealed that blocking CD47 inhibits the growth of ATCs and reduces TAMs density, thereby suppressing tumor growth (Schürch et al., 2019). Caluo and his colleagues identified a specific type of TAM in ATC, called “ramified TAM” (RTAM), which is not present in other types of TCs, and forms a high dense network interlinked with tumor cells in ATC, providing signals and nutrients for tumor growth (Caillou et al., 2011).

### Functions of M2-Macrophages in Medullary Thyroid Cancer and Follicular Thyroid Cancer

A recent *in vitro* study showed that CCL15 derived from FTC cells can effectively recruit TAMs to TME, thus providing a favorable microenvironment for tumor growth (Huang et al., 2016). MALAT1-mediated TAMs secretes FGF2 (bFGF) to inhibit the release of inflammatory cytokines, promote the proliferation, migration and invasion of FTC133 cells, and induce angiogenesis (Huang et al., 2017). In transgenic mouse models of FTC, a mechanism was found that advanced cancer in emasculated men was due to increased expression of tumor suppressor genes:GLIPR1 and SFRP1, resulting in increased tumor invasion of M1-macrophages and CD8 cells. In addition, GLIPR1 slows down the growth of cancer cells and increases the secretion of CCL5, which leads to the activation of immune cells (Zhang et al., 2015). However, studies on TAMs in FTC is still lacking due to tumor samples limitations, and large samples of clinical and basic studies are needed to confirm this function. Interestingly, there is still no study or report on MTC and TAMs, and the relationship between the two is still unclear.

## Macrophage-Derived Cytokines and Chemokines Interact in Thyroid Cancer

### Macrophage-Derived Cytokines in Thyroid Cancer

TAMs not only provide structural support for tumor growth, but also participate in tumor development by secreting signaling molecules (Kogure et al., 2019). Although TAMs have insufficient secretory function, they can secrete cytokines or directly stimulate TC cells. Though some of the macrophage phenotypes will have anti-tumor immune function, but in recent years, there are evidence that TAMs can reconstruct TME, promote tumor cell proliferation and survival, promote angiogenesis and lymphatic vessel formation, and suppress T cell response to tumors (Mantovani and Allavena, 2015).

TAMs can also increase angiogenic factors to promote tumor angiogenesis. A recent study confirmed that TAMs promote tumor angiogenesis by upregulating VEGF (Hughes et al., 2015; Osterberg et al., 2016). In addition, VEGF secreted by TAMs increased the growth and activity of tumor microvasculature, providing suitable microenvironment for TC infiltration and metastasis. Examples include VEGF, platform-derived growth factor (PDGF), bFGF (Melaccio et al., 2021). In addition, it can also produce the matrix metalloproteases (MMPS) (Ojalvo et al., 2010; Cheng et al., 2021). Angiogenesis is related to tumor growth and metastasis and plays an important role in tumor development. Therefore, TAMs are one of the important factors in tumor angiogenesis (Chen et al., 2019).

In a BRAF^V600E^ mutation-induced mouse tumor model, TC cells and TAMs secrete TGF-β, which leads to tumorigenic EMT and increases the invasiveness of TC cells (Knauf et al., 2011). TAMs not only secretes some cytokines to promote the EMT of TC, but also maintains the stemness of TC. In TC, IL-6 is dependent on the activation of the IL6/JAK1/STAT3 signaling pathway to promote thyroid CSC proliferation and colony formation and to enhance the properties of thyroid CSCs and EMT (Zheng et al., 2019). IL-10 is secreted by both TAMs and malignant tumor cells. IL-10 has various effects in the immune system, most notably in the form of immunosuppression. IL-10 is found to be overexpressed in TCs and its expression was shown to be correlated with tumor size and tumor extension, suggesting that IL-10 might enhance the function of tumor progression (Mocellin et al., 2005; Cunha et al., 2017).

In summary, after monocytes are recruited near the tumor and differentiate into TAMs, TAMs secrete a variety of molecules, including growth factors, cytokines, and proteases, that promote tumor angiogenesis, immunosuppression, and tumor metastasis.

### Macrophage-Derived Chemokines in Thyroid Cancer

Chemokines are classified into four subtypes: C, CC, CXC, and CX3C, and CC in the predominant one of these subtypes (Yapa et al., 2017). The role of cytokines is to create a concentration gradient to induce motility or chemotaxis for specific receptor cells, inducing the migration of immune cells such as NK-cells, dendritic cells, and others (Mukaida et al., 2014). Previous studies have identified a variety of chemokine-mediated immune cells that promote progression of TCs (Yapa et al., 2017). However, the signaling pathways by which these chemokines mediate immune cells are diverse. TC cells or normal thyroid cells secrete a variety of chemokines in the basal state or under stimulation (Mironska et al., 2019).

In the studies on chemokine-mediated macrophages, cellular CXCL8 was the first chemokines found to be associated with TC, and cell lines from PTC and ATC were found to contain high levels of CXCL8 and TAMs, leading to tumor proliferation and metastasis (Kobawala et al., 2011). When CXCL8 is blocked, the metastatic potential of PTC is significantly reduced, and conversely, increasing exogenous CXCL8 promotes the metastatic potential of PTC (Fang et al., 2014). In addition, Visciano and his colleagues used *in vitro* cell culture to demonstrate that (IL-8) CXCL8 induces EMT in TC and enhances the stemness of thyroid CSCs through the Akt-Slug signaling pathway (Visciano et al., 2015). These chemokines also have the function of promoting EMT in TC (Pradella et al., 2017).

In a study of another chemokine receptor, CXCR4, whose ligand is CXCL12, activates G protein-mediated signaling pathways, including AKT, JAK/STAT, and MAPK (Pawig et al., 2015), and promotes migration and invasion of a variety of malignancies, including TCs. High expression of CXCR4 promotes cell proliferation and lymph node metastasis in PTCs and ATCs (Hwang et al., 2003; Wagner et al., 2008). In addition, there are many chemokines that can enhance the proliferation and invasion of TC. For example, CCL2/MCP-1 (monocyte chemotactic protein-1) promotes lymph node metastasis in PTC by recruiting TAMs expressing CCR2, and patients with high CCL2 expression are more likely to recurrence (Tanaka et al., 2009). The chemokine CCL21 and its receptor CCR7 were found to play crucial roles in the proliferation and migration of PTC and MTC by Sancho et al. (2006). In PTC, patients with elevated CCL2 are more likely to develop lymph metastases and have a high recurrence risk (Tanaka et al., 2009). In another experiment found that PTC overexpression of CXCL16 is correlated with M2 polarization and promote tumor angiogenesis (Kim et al., 2019). A recent study confirmed that high CXCR6 expression was positively associated with PTC lymph metastasis and that CXCL16 mediated macrophages invasion of PTC and altered the macrophages phenotype to M2-TAMs in the TME (Cho et al., 2016).

## Targeting Tumor-Associated Macrophages Is a Potential Treatment for Thyroid Cancer

For all types of TC, surgical treatment is still the best means of treatment, and TSH inhibition therapy could be performed according to the postoperative pathological results such as lymph node metastasis of patients and the risk of recurrence (Haugen et al., 2015). If necessary, ^131^I therapy, external radiation or targeted therapy should be performed (Wang et al., 2019b). Tumor immunotherapy is an effective anti-tumor therapy strategy developed in recent years. There is considerable evidence that macrophages are polarized into the M2 phenotype in the development of many solid tumors (Komohara et al., 2016; Movahedi and Van Ginderachter, 2016). Therefore, targeting TAMs is a potential therapeutic strategy for solid tumors. The transformation of M2 to M1-macrophages is a potential new antitumor immunotherapy, and its mechanism is related to the upregulation of macrophage phagocytosis. Current strategies for macrophage therapy are mainly through two approaches: inhibition of recruitment and/or clearance, and reversal of differentiation. Blocking macrophages recruitment has been extensively studied in preclinical models, and is being evaluated for feasibility of clinical application. Several monoclonal antibodies that have been approved for clinical use in the treatment of tumors have been shown to work therapeutically, primarily by increasing the phagocytic activity of macrophages. Targeted regulation of TAMs plays a key role in the activity of antitumor drugs (Germano et al., 2013).

### Transform or Inhibit the Polarization of M2-Macrophages

Inhibition of cytokines or chemokines, such as CCL2 and colony stimulating factor (CSF-1), that promote the polarization of macrophages into M2 phenotype, is a promising immunotherapy (Kim et al., 2013; Richards et al., 2013). Tyrosinase inhibitors for TC and immunotherapy drugs under development are listed in Table 1. A recent study showed that PLX4720 (The main active molecule is 7-azaindole, which is a potent and selective inhibitor of B-Raf V600E) combined with anti-PD-L1/PD-1 antibody significantly reduced ATC tumor volume, increased the density and cytotoxicity of CD8^+^ T cells and NK cells, increased M1-TAMs, prolonged survival, improved TME, and enhanced anti-tumor immune effect (Gunda et al., 2018). In addition, Rituximab is a chimeric monoclonal antibody that specifically binds to the transmembrane antigen CD20 and inhibit the growth of non-Hodgkin’s lymphoma cells by promoting macrophage phagocytosis (Chao et al., 2010). Trastuzumab blocks the attachment of human EGF to Her2 by attaching itself to Her2 and mediate macrophage killing of Her-2 overexpressing in breast cancer cells (Shi et al., 2015). Similarly, bleomycin mainly interferes with DNA synthesis, but low dose bleomycin can reverse the M2 to M1-macrophages, and the proliferation, migration and invasion of TPC-1 cells are significantly reduced, suggesting that bleomycin can inhibit the progression of PTC by modulating macrophages polarization (Liu et al., 2018b). Similarly, selective elimination of M2-TAMs inhibits PTC growth, and CSF-1 signaling may serve as a potential therapeutic target for inhibiting BRAF^V600E^ mutation-induced PTC (Ryder et al., 2013). More interestingly, A bisphosphonate (zoledronic acid) binds to microcalcification in tumor tissue and is subsequently phagocytized by TAM to induce apoptosis, and also promotes polarization of M2 towards M1-macrophages (Hattori et al., 2015). A mouse model of combined anti-macrophage zoledronic acid and tumor cytotoxic docetaxel showed that the combined strategy significantly inhibited tumor growth and pulmonary metastasis (Sun et al., 2015). Blocking and targeting the CCL-2/CCR2 and CSF-1/CSF-1R pathways is a promising approach in ATC. This approach can not only inhibit the recruitment of tumor M2-macrophages, but also re-polarize them into the M1 phenotype (Naoum et al., 2018).

**TABLE.1:** Tyrosine kinase inhibitors and new immunotherapeutics in development for thyroid cancer.

Drug	Therapeutic target	Indication	Dose	Results	Research progress	Reference
Dabrafenib plus Trametinib	BRAF and MEK	ATC	150 twice daily and 2 mg once daily	63% PR	In clinical trial	Subbiah et al. (2018)
Selpercatinib	RET	MTC	160 twice daily	61% PR	Approved for clinical use	Wirth et al. (2020)
Anlotinib	VEGF, VEGFR, PDGFR, c-Kit, RET	MTC	12mg once daily	56.9% PR	Approved for clinical use	Sun et al. (2018)
Pralsetinib	RET	MTC	400 mg once daily	91%	Approved for clinical use	Markham (2020)
Vandetanib	RET, VEGF, VEGFR	MTC	300 mg once daily	45% PR	Approved for clinical use	Koehler et al. (2021)
Cabozantinib	RET, VEGFR, c-MET	MTC	140 mg once daily	28% PR	Approved for clinical use	Koehler et al. (2021)
Axitinib	VEGF, VEGFR	ATC	5 mg twice daily	30% PR	In clinical trial	Cohen et al. (2014)
Lenvatinib	VEGF, VEGFR, FGFR, RET, c-kit	ATC/MTC	24 mg daily	24%/36% PR	Approved for clinical use	Tahara et al. (2017)
Pazopanib	VEGFR, PDGFR, FGFR, c- kit	MTC	800 mg daily	14% PR	In clinical trial	Bible et al. (2014)
Sorafenib	VEGFR, PDGFR, RET, c-kit, BRAF	MTC	400 mg twice daily	24% PR	Approved for clinical use	Lam et al. (2010)
Sunitinib	VEGFR, GIST, PDGFR, RET, c-kit, CSF-1R, Flt-3	MTC	37.5 mg daily	24% PR	In clinical trial	Carr et al. (2010)

Immunotherapy strategies and therapeutic targets for thyroid cancer under development.							
Drug	Therapeutic target	Research progress	Indication	Drug	Therapeutic target	Research progress	Indication
LY3022855	CSF-1R, TAM	In clinical trial	TC	Cemiplimab	PD-1, PD-L1	In clinical trial	ATC
PLX3397 plus Paclitaxel	CSF-1R, TAM	In clinical trial	TC	MLN0128(mTORi)	mTOR1/2	In clinical trial	ATC
LY3022855 plus Tremelimumab or Durvalumab	CSF-1R, TAM, PD-1	In clinical trial	TC	Pembrolizumab	PD-1, PD-L1	Approved for clinical use	ATC
PLX3397 plus Pembrolizumab	CSF-1R, TAM, PD-1	In clinical trial	TC	Trametinib plus Paclitaxel	MEK	In clinical trial	ATC

### Blocking the Secretion of Cytokines/Chemokines or Their Receptors in the TME

Blocking common TAM-related chemokines such as CCL2 and CXCL8 and their associated receptors may also be a potential therapeutic strategy. Since ELR- can counteract the effect of ELR+, targeting ELR + CXC chemokines can also inhibit tumorigenesis (Mukaida et al., 2014). Stassi et al. found that IL-4 and IL-10 promoted the progression of TC cells and resistance to chemotherapy through upregulation of anti-apoptotic proteins. Therefore, IL-4 and IL-10 may be new therapeutic targets for TC (Stassi et al., 2003). In addition, TC cells have some functional receptors that receive signals of chemokines for proliferation, immunosuppression, angiogenesis and other activities to maintain the growth and metastatic potential of tumor cells (Mantovani et al., 2010; Allavena et al., 2011). In a BRAF^V600E^ mutant PTC mouse model, IL-12 treatment significantly reduced tumor size and weight and improved OS (Parhar et al., 2016). Another study identified IL-12 as a pro-inflammatory cytokine with anti-tumor activity, demonstrating inhibition of ATC growth (Lu, 2017). Possible molecular mechanisms are associated with NF-κB activation and NF-κ B-dependent MMP-3 upregulation. Therefore, molecular therapies targeting CCL20 and CCR6 may provide promising intervention strategies for TC (Cunha et al., 2012; Zeng et al., 2014). In a animal model experiment, the change of secretory CXCL8 in the treatment of MTC can be used as a biomarker for the clinical efficacy of sunitinib (Broutin et al., 2011). In addition to MTC and ATC, the prognosis of PTC is also closely related to TME. Researchers are looking for an immunotherapy to modify TME to enhance the outcome of surgery and RAI (Na and Choi, 2018). Balkwill et al. demonstrated that CXCR7, when downregulated in PTC, inhibits cell growth and invasion, leads to S-phase arrest, and promotes apoptosis, suggesting that CXCR7 may be a promising target for therapeutic intervention of PTC (Balkwill, 2012).

The plasticity of macrophages highlights the potential of macrophage reprogramming as a therapeutic strategy to inhibit tumor progression, enabling these cells to adapt their functions to meet the needs of tumor resistance. In order to better understand the activation status of TAMs in TME, further studies on specific markers are needed to distinguish the different functions of antitumor or pro-tumor TAMs.

## Conclusion and Perspectives

In recent decades, there has been no significant improvement in the survival of patients with progressive or recurrent TCs, despite systematic and multidisciplinary treatments. Therefore, for the postoperative management of TC patients, in addition to histological classification, other pathological parameters such as mutational status, activation of molecular signaling pathways, tumor cell differentiation, and associated immunophenotype need to be considered. TME plays an important role in TC development, metastasis, stemness, and TAMs account for the largest proportion of cells in TME. Although it is currently believed that high infiltration of M2-TAMs supports the TME and promotes the growth of TC, it has also been suggested that mixed immune cell infiltration may be associated with a good prognosis in differentiated TC. The study of TAMs in TC is a novel field and more research is needed to unravel the complex and dynamic crosstalk between TC cells and their microenvironment. Although current research has evidence to support that targeting TAMs can significantly improve the efficacy of conventional therapies and immunotherapy, there is still a long way to go to understand the role and mechanisms of TAMs in TC progression and the use of TAM-based immunotherapy.
